# Activity of Semi-Synthetic Mulinanes against MDR, Pre-XDR, and XDR Strains of *Mycobacterium tuberculosis*

**DOI:** 10.3390/metabo11120876

**Published:** 2021-12-16

**Authors:** María Alejandrina Martínez-González, Luis Manuel Peña-Rodríguez, Andrés Humberto Uc-Cachón, Jorge Bórquez, Mario J. Simirgiotis, Hugo Brígido Barrios-García, Rogelio Hernández-Pando, Luis Alberto Loyola, Carlos Areche, Angel de Jesús Dzul-Beh, Jorge Alberto Barrios-Payán, Dulce Mata-Espinosa, Fabiola Escalante-Erosa, Karlina García-Sosa, Gloria María Molina-Salinas

**Affiliations:** 1 Unidad de Investigación Médica Yucatán, Unidad Médica de Alta Especialidad, Hospital de Especialidades 1, Instituto Mexicano del Seguro Social, Merida 97150, Yucatan, Mexico; malejandrinamg_16@hotmail.com (M.A.M.-G.); andresuccachon@gmail.com (A.H.U.-C.); angeldzulbeh1992@gmail.com (A.d.J.D.-B.); 2 Unidad de Biotecnología, Centro de Investigación Científica de Yucatán, A.C., Merida 97205, Yucatan, Mexico; fabiola@cicy.mx (F.E.-E.); karlina@cicy.mx (K.G.-S.); 3 Departamento de Química, Facultad de Ciencias Básicas, Universidad de Antofagasta, Antofagasta 02800, Chile; jorge.borquez@uantof.cl (J.B.); mario.simirgiotis@gmail.com (M.J.S.); alberto.loyola@uantof.cl (L.A.L.); 4 Facultad de Ciencias, Instituto de Farmacia, Universidad Austral de Chile, Valdivia 3425, Chile; 5 Facultad de Medicina Veterinaria y Zootecnia, Universidad Autónoma de Tamaulipas, Ciudad Victoria 87000, Tamaulipas, Mexico; hbarrios@docentes.uat.edu.mx; 6 Departamento de Patología Experimental, Instituto Nacional de Ciencias Médicas y Nutrición Salvador Zubirán (INCMNSZ), Ciudad de Mexico 14080, Mexico; rhdezpando@hotmail.com (R.H.-P.); qcjbp77@yahoo.com.mx (J.A.B.-P.); dulmat@yahoo.com.mx (D.M.-E.); 7 Departamento de Química, Facultad de Ciencias, Universidad de Chile, Santiago 1058, Chile; areche@uchile.cl

**Keywords:** MDR, pre-XDR and XDR tuberculosis, *Mycobacterium tuberculosis*, semi-synthetic derivatives, mulinanes

## Abstract

Tuberculosis causes more than 1.2 million deaths each year. Worldwide, it is the first cause of death by a single infectious agent. The emergence of drug-resistant strains has limited pharmacological treatment of the disease and today, new drugs are urgently needed. Semi-synthetic mulinanes have previously shown important activity against multidrug-resistant (MDR) *Mycobacterium tuberculosis*. In this investigation, a new set of semi-synthetic mulinanes were synthetized, characterized, and evaluated for their in vitro activity against three drug-resistant clinical isolates of *M. tuberculosis*: MDR, pre-extensively Drug-Resistant (pre-XDR), and extensively Drug-Resistant (XDR), and against the drug-susceptible laboratory reference strain H37Rv. Derivative 1a showed the best anti-TB activity (minimum inhibitory concentration [MIC] = 5.4 µM) against the susceptible strain and was twice as potent (MIC = 2.7 µM) on the MDR, pre-XDR, and XDR strains and also possessed a bactericidal effect. Derivative 1a was also tested for its anti-TB activity in mice infected with the MDR strain. In this case, 1a produced a significant reduction of pulmonary bacilli loads, six times lower than the control, when tested at 0.2536 mg/Kg. In addition, 1a demonstrated an adjuvant effect by shortening second-line chemotherapy. Finally, the selectivity index of >15.64 shown by 1a when tested on Vero cells makes this derivative an important candidate for future studies in the development of novel antitubercular agents.

## 1. Introduction

Tuberculosis (TB) is a communicable disease caused by the *Mycobacterium tuberculosis* complex that is a major cause of illness. TB is one of the top 10 causes of death worldwide, and the leading cause of death from a single infectious agent. In 2019, an estimated 10 million people fell ill with TB worldwide and there were 1.2 million TB deaths among HIV-negative individuals, with the addition of 208,000 deaths among HIV-positive patients [1].

The TB epidemic has been aggravated by the emergence of drug-resistant strains of *M. tuberculosis*, including multidrug-resistant (MDR; mycobacteria resistant to the first-line anti-TB pharmaceuticals Isoniazid and Rifampicin), pre-eXtensively Drug-Resistant (pre-XDR; MDR strains, which are also resistant to any fluoroquinolone), and eXtensively Drug-Resistant (XDR; MDR and that are also resistant to any fluoroquinolone and at least one drug from Group A (that includes some of the most potent pharmaceuticals for the treatment of drug-resistant forms of TB, e.g., Levofloxacin, Moxifloxacin, Bedaquiline, and Linezolid) [2,3]. Worldwide, drug-resistant TB continues to be a public health threat, with nearly half a million patients developing Rifampicin resistance, and with 78% of these individuals showing MDR-TB [1]. By the end of 2018, at least one case of XDR-TB was reported by each of the 131 World Health Organization (WHO) Member States, and data suggest that about 6.2% of MDR-TB cases worldwide have XDR-TB [4]

Presently, anti-TB treatment requires combinations of several drugs, ranging from a period of 6 months for drug-susceptible TB, to 9–20 months for MDR-TB, or longer if there is additional drug resistance, such as pre-XDR or XDR-TB. The key challenges in the treatment of TB are the duration and complexity of the regimens, both of which affect treatment adherence by the patient, and toxic side-effects, especially of drugs used to treat drug-resistant TB. In addition, drug–drug interactions have been reported between anti-TB drugs and antiretroviral therapies for persons living with HIV [5]. Currently, there is a pressing need for new anti-TB drugs that are more effective against drug-resistant TB, while being nontoxic, affordable, and requiring a short treatment regime. The mulinane diterpenoids comprise a group of natural products isolated exclusively from South American flowering shrubs of the *Mulinum, Azorella*, and *Laretia* genera, mainly from *Azorella compacta* Phil and *Mulinum crassifolium* Phil [6,7]. These medicinal plants have been used in traditional folk medicine to treat colds, pain, diabetes, asthma, bronchitis, womb ailments, gastric disorders, backache, wounds, and altitude sickness [8]. Structurally, the mulinanes have a tricyclic skeleton of fused five-, six-, and seven-membered rings, with an angular substituent at each of the ring junctions. Nearly all mulinanes are characterized by having a carboxyl group at the C-20 position and a functionalized seven-member ring [7,9]. In addition, mulinanes have been reported to show a wide variety of biological activities including antimicrobial, antiprotozoal, antitumor, anti-inflammatory, and antimycobacterial, among others [7].

We have previously reported several semi-synthetic alkylated mulinanes, which showed promising in vitro activity against MDR *M. tuberculosis* [10]. As part of our continuing search for novel bioactive derivatives against MDR, pre-XDR, and XDR TB, we wish to report on the preparation of a new series of semi-synthetic mulinanes and the results of their in vitro and in vivo evaluation against drug-resistant *M. tuberculosis* strains.

## 2. Results

### 2.1. Purification and Chemical Transformation of Mulinanes

**Figure 1:**
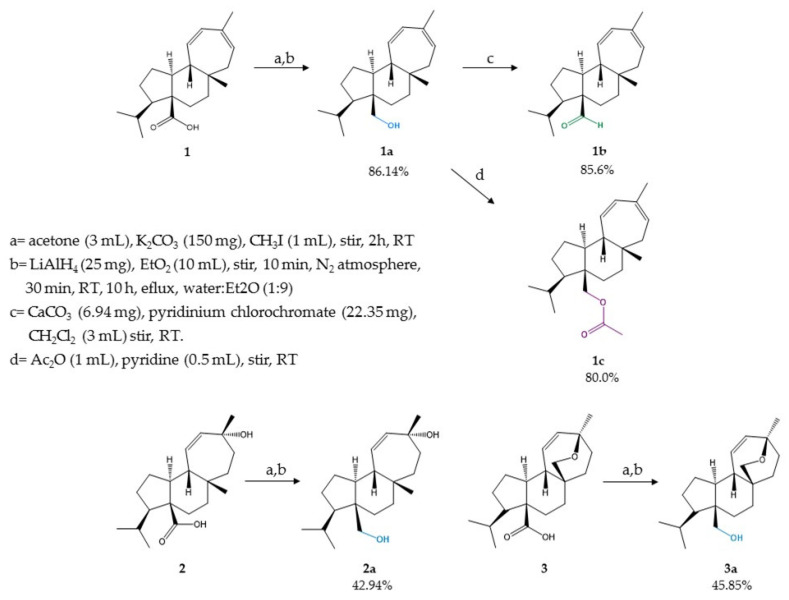
Preparation of semisynthetic derivatives from the natural mulinanes: mulin-11,13-dien-20-oic acid (1); mulin-11,13-dien-20-ol (1a); mulin-11,13-dien-20-al (1b); mulin-11,13-dien-20-yl acetate (1c); 13α-hydroxymulin-11-en-20-oic acid (2); 13α-hydroxymulin-11-en-20-ol (2a); mulinenic acid (3); mulinenol (3a).

Semi-synthetic derivatives of natural mulinanes were prepared as shown in Figure 1.

The chemical structure of the semi-synthetic mulinanes was confirmed by their ^1^H- and ^13^C-NMR, IR, and MS data. The complete set of natural and semi-synthetic mulinanes were tested in vitro for anti-TB and cytotoxic activity, and the most active semi-synthetic mulinane was further evaluated in an anti-TB murine model. 

### 2.2. In Vitro Antituberculosis Activity and Lipophilicity of Mulinanes

Natural and semi-synthetic mulinanes (Figure 1) were evaluated using the modified Microplate Alamar Blue Assay (MABA) against clinical isolates of MDR, pre-XDR, and XDR strains of *M. tuberculosis*, together with the susceptible strain H37Rv (ATCC 27294) as a reference (Table 1) [11]. The minimum inhibitory concentration (MIC), minimum bactericide concentration (MBC), and lipophilicity of natural and semi-synthetic mulinanes are shown in Table 1. Interestingly, the MIC and MBC value of all natural and semi-synthetic mulinanes is the same. 

### 2.3. Cytotoxicity and Selective Index of Mulinanes

The evaluation of the cytotoxic activity and selective index (SI) of the natural and semi-synthetic mulinanes is reported in Table 2.

### 2.4. In Vivo Antituberculosis Activity of Semi-Synthetic Mulinane 1a

**Figure 2:**
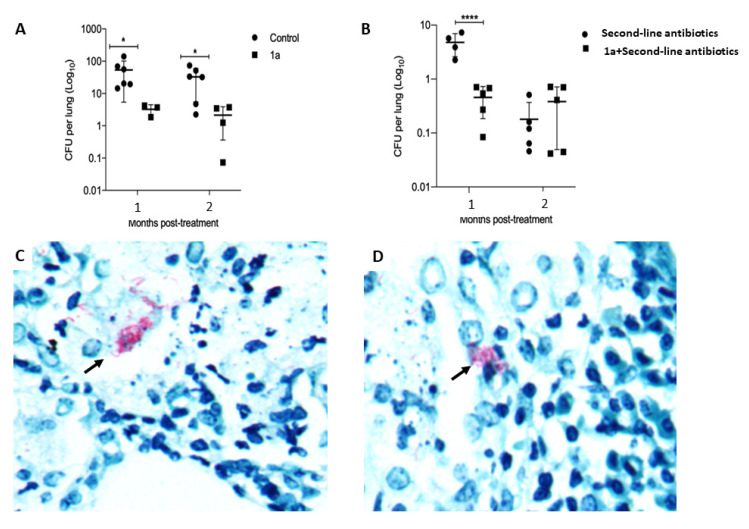
Therapeutic effect of semi-synthetic mulinane 1a in BALB/c mice at 60 days post-infection with the MDR *M. tuberculosis* strain. (**A**) Groups of mice were treated with 1a (▪), while control animals received only the vehicle (•); the mice were euthanized after one and two months of treatment, and lungs were used for the determination of mycobacterial load by Colony-Forming Units. The treatment with 1a produced a significant decrease of bacillary loads. (**B**) Lung bacillary loads after treatment with 1a and second-line antibiotics (▪) in comparison with infected mice treated only with second-line antibiotics (•). After one month of treatment, the 1a plus second-line antibiotic regimen produced a significant decrease of bacilli burdens compared to the mice treated with only second-line antibiotics. The results are expressed as the mean ± standard deviation of three independent experiments with three different animals at each point-of-sacrifice. Asterisks represent statistical significance (**** *p* < 0.0001, * *p* < 0.05, two-way ANOVA). Representative high-power micrographs of lung sections stained with Ziehl-Neelsen: (**C**) larger clusters of bacilli (arrow, red rods) in control untreated mouse compared to in mouse treated for two months with 1a (**D**).

Animals treated with 1a exhibited a significant (six-fold) reduction of pulmonary bacilli loads, when compared to those of the control mice (Figure 2A). Ziehl-Neelsen-stained sections from mice treated with 1a showed smaller clusters of bacilli than control untreated mice (Figure 2C,D). To determine if mulinane 1a could be useful in shortening the duration of second-line chemotherapy, 60 days after mice were infected with the MDR TB strain, treatment with second-line antibiotics alone or in combination with 1a was initiated. After one month of treatment with 1a and second-line antibiotics, a significant reduction in bacillary burden was observed when compared with mice that received only second-line antibiotics (Figure 2B).

## 3. Discussion

### 3.1. Chemical Transformation of Mulinanes

Direct reduction of the carboxyl group of natural diterpenoids 1, 2, and 3 could not be accomplished using LiAlH_4_, because of the low reactivity of the carboxyl group. However, a reduction of the corresponding methyl esters obtained through treatment of 1, 2, and 3 with iodomethane, an inexpensive good-yield-producing reagent [12], allowed the preparation of the reduced derivatives 1a, 2a, and 3a.

Controlled oxidation of the hydroxyl group of 1a to produce the corresponding aldehyde (1b) was carried out using a Corey–Suggs oxidation with pyridinium chlorochromate [13]. Alternatively, esterification of 1a using the Fischer method yielded the corresponding acetate 1c.

### 3.2. In Vitro Antituberculosis Activity and Lipophilicity of Mulinanes

The results showed the best in vitro anti-TB activity for the semi-synthetic derivative mulin-11,13-dien-20-ol (1a) (Table 1), with an MIC value of 5.4 µM against the susceptible reference strain, and an activity twice as strong (MIC = 2.7 µM) against the resistant strains MDR, pre-XDR, and XDR. The lower anti-TB activity shown by the oxidized and esterified derivatives 1b and 1c, respectively, suggests that complete reduction of the carboxyl group to an unesterified primary alcohol is important for the expression of anti-TB activity [14]. These results, and the importance of the primary alcohol at C20 for the expression of anti-TB activity, were confirmed when the semisynthetic derivatives 2a and 3a proved to be more active than their natural precursors [15].

The antimycobacterial activity of semi-synthetic mulinanes 1a, 1b, 2a, and 3a against strains of *M. tuberculosis* resistant to either first- or second-line drugs can be considered particularly important since the Clinical and Laboratory Standards Institute has established that pure compounds with antimycobacterial activity with MIC values below 25 µM are relevant [14]. Additionally, these semi-synthetic mulinanes showed the same MBC and MIC values in all *M. tuberculosis* strains, indicating that their inhibitory growth concentration is mycobactericidal too. Furthermore, previous results have shown that methyl ester derivatives of structurally related semi-synthetics were also active anti-TB mulinanes (MIC = 12.5–25 μg/mL) [10], and their activity increased when preparing the corresponding *n*-propyl and *n*-butyl esters (MIC = 6.25 μg/mL) [15]. Since the anti-TB activity of 1a (MIC = 0.78–1.56 μg/mL) is the best registered to date, 1a can be considered as an important lead molecule in future semi-synthetic or biotransformation studies to improve its anti-TB activity.

Even though the lipophilicity of natural and semi-synthetic derivatives has been reported to be important for favoring their penetration through the lipophilic mycobacterial cell walls [16], no direct correlation was observed between the calculated lipophilicity (logP) and the antimycobacterial activity (Table 1) of the different natural mulinanes and their semi-synthetic derivatives, where the most lipophilic semi-synthetic derivative 1c (logP = 7.34 ± 0.29) proved to be the least active against TB, while the rest of the mulinanes, natural and semi-synthetic, showed similar lipophilicity values but significant differences in their anti-TB activity. While these findings suggest that lipophilicity does not appear to play a significant role in the anti-TB activity of the natural or semisynthetic mulinanes, they do not coincide with results reported in the literature describing that an increase in the lipophilicity of a series of pyridines increased their anti-TB activity [17], and with similar results obtained when evaluating the anti-TB activity of lipophilic derivatives of diphenyl- and diaryl-pyrroles [18,19,20], and of pentacyclo-undecane-derived cyclic tetraamines [21].

### 3.3. Cytotoxicity and SI of Mulinanes

The evaluation of the cytotoxic activity of the natural and semi-synthetic mulinanes showed SI values of 15.64, 64, and 64 for the most anti-TB active semi-synthetic derivatives 1a, 2a, and 3a, respectively, with derivatives 2a and 3a not being cytotoxic to the Vero cell line at concentrations of >624 µM. These results, which are particularly relevant when taking into account that promising antibacterial agents are recommended to have an SI value of >10 [22], suggest that semi-synthetic derivatives 1a, 2a, and 3a have high biomedical potential for their future development into antimycobacterial agents against drug-resistant *M. tuberculosis*. 

### 3.4. In Vivo Antituberculosis Activity of Compound 1a

To evaluate whether treatment with 1a can help to shorten the duration of the second-line anti-TB chemotherapy regime, a group of MDR-infected mice were treated, after 60 days post-infection, for two months with a combination of 1a and a regime of second-line antibiotics and compared with a group of infected animals that received only antibiotics. The results showed that animals that received the combined treatment demonstrated a significant six-fold reduction of bacillary burdens after one month of treatment, when compared to mice treated only with antibiotics (Figure 2B). This rapid reduction of pulmonary bacilli loads as a consequence of the combined treatment suggested that the semi-synthetic derivative 1a could be used as adjuvant to shorten second-line chemotherapy.

## 4. Materials and Methods

### 4.1. General Procedures and Chromatographic Techniques

All starting materials, chemicals, and solvents were purchased from commercial suppliers (Sigma-Aldrich, St. Louis, MO, USA; Merck, Darmstadt, Germany; Becton Dickinson, NJ, USA). All technical-grade solvents employed for chromatographic separations were distilled prior to use. Solvents were evaporated utilizing a rotary evaporator (Büchi, Flawil, Switzerland).

Silica gel 60 (Merck, Darmstadt, Germany) was used for column chromatography (CC), and pre-coated silica gel plates (Merck, Kieselgel 60 F254, 0.25 mm) were used for preparative thin-layer chromatography (TLC). Detection in TLC was achieved under ultraviolet (UV) light at 254 and 365 nm. Visualization of components was carried out by spraying with a phosphomolybdic acid reagent (Sigma-Aldrich, St. Louis, MO, USA), followed by heating for 5 min at 105 °C.

### 4.2. Spectroscopic and Spectrometric Techniques

^1^H- and ^13^C-NMR spectra were recorded using a Bruker Avance 400 (Burker Co., Billerica, MA, USA) operating frequency 400 MHz for ^1^H and 100.61 MHz for ^13^C). Deuterated chloroform (CDCl_3_, Sigma-Aldrich, St. Louis, MO, USA) was used as solvent and tetramethylsilane (TMS, Sigma-Aldrich, St. Louis, MO, USA) as an internal reference. Chemical shift values are quoted in parts per million (ppm, δ), and coupling constants (*J*) are quoted in Hertz (Hz). Infrared spectra were taken on KBr discs on a FT-IR Nicolet Magna 750 spectrophotometer (Nicolet Company, Madison, WI, USA). EIMS were obtained on Agilent Technologies 6890N (Agilent Technologies, Santa Clara, CA, USA). The gas chromatograph was coupled to an Agilent Technologies 5975B (Agilent Technologies, Santa Clara, CA, USA) inter-mass selective detector.

### 4.3. Isolation and Purification of Natural Mulinanes

Mulin-11,13-dien-20-oic acid (1), 13-α-hydroximulin-11-en-20-oic acid (2), and mulinenic acid (**3**) were obtained from fractions of *Azorella compacta* and *Mulinum crassifolium* collected in October 2013 in El Tatio, in the mountains of the High-Andean steppe of region II of Chile at 4300 m above sea level (m.a.s.l.), as previously reported by Dzul-Beh et al. in 2019 [23]. The dried and ground plant material was successively extracted by percolation at room temperature (RT) with solvents of different polarity (hexane [Hex], ethyl acetate [EtOAc], and methanol). Extraction solvents were concentrated under reduced pressure in a rotary evaporator (40 °C) and the resulting hexane extracts of both species were subjected to VLC purification using silica gel and a gradient elution with mixtures of Hex:EtOAc. Relevant fractions were purified by successive open-CC on silica gel, using a gradient elution with mixtures of Hex:EtOAc, until the pure diterpenes were obtained. The purified metabolites were identified by comparing their spectroscopic data with those reported in the literature [24,25,26].

### 4.4. Preparation of Semi-Synthetic Mulinane Derivatives

The preparation of 2a (20.5 mg [42.94%]) and 3a (22mg [45.85%]) was carried out following the same experimental procedure as that described for the preparation of mulin-11,13-dien-20-ol (1a) using 2 and 3 as starting materials, respectively. A sample of mulin-11,13-dien-20-oic acid (1, 50 mg), K_2_CO_3_ (150 mg), CH_3_I, (1 mL), and acetone (3 mL) was added to a 25-mL round-bottom flask and the solution was stirred at RT for 2 h. Water (14 mL) was then added to the reaction mixture, and the aqueous layer was extracted with EtOAc (2 × 4.5 mL). The organic layer was dried over anhydrous Na_2_SO_4_, and then the organic solvent was removed under reduced pressure to yield a crude methyl ester, which was added (68 mg in 2 mL of anhydrous Et_2_O) in a three-neck round-bottom flask under N_2_ atmosphere, dropwise, to a mixture of an excess of LiAlH_4_ (25 mg) and anhydrous ethyl ether (Et_2_O, 10 mL), which was previously stirred for 10 min in a three-neck round-bottom flask under a N_2_ atmosphere. The reaction mixture was allowed to stir for 30 min at RT and then under reflux for 10 h. After cooling to RT, the reaction mixture was quenched by adding a 1:9 mixture of water and Et_2_O (1:9) dropwise, followed by EtOAc (5 mL) and MeOH (1 mL). The organic layer was separated, dried over anhydrous Na_2_SO_4_, and evaporated to dryness to yield 17 mg (86.14%) of pure mulin-11,13-dien-20-ol (1a) as a colorless wax after purification by multiple elution (eluted twice) preparative TLC using Hex:EtOAc (9:1) as the eluted. The spectroscopic and spectrometric data of 1a, 2a, and 3a are shown in Table S1. 

Mulin-11,13-dien-20-al (1b): a sample of mulin-11,13-dien-20-ol (1a, 20 mg), CaCO_3_ (6.94 mg), pyridinium chlorochromate (22.35 mg), and CH_2_Cl_2_ (3 mL) were added to a 25-mL balloon flask and the solution was stirred at RT until total consumption of 1a was observed by TLC. The mixture was diluted with Et_2_O (10 mL) and then passed through a silica gel (70–230 mesh) column. Purification of the crude oxidation product by preparative TLC using Hex:EtOAc (9:1, 2×) as the eluent yielded 17 mg (85.60%) of mulin-11,13-dien-20-al (1b) as a yellow oil. The spectroscopic and spectrometric data of 1b are shown in Table S2. 

Mulin-11,13-dien-20-yl acetate (1c): a sample of mulin-11,13-dien-20-ol (1a, 15 mg), Ac_2_O (1 mL), and pyridine (0.5 mL) were combined in a 25-mL balloon flask and the mixture was stirred at RT until total consumption of 1a was observed by TLC. The reaction mixture was diluted with 10 mL of distilled water and the aqueous layer was extracted with CH_2_Cl_2_ (2 × 10 mL). The organic layer was washed with an aqueous solution of 5% HCl, dried over anhydrous Na_2_SO_4_, and evaporated to dryness. The crude esterification product was purified by preparative TLC using Hex:EtOAc (9:1, 2×) as the eluent, obtaining 13 mg (80.00%) of mulin-11,13-dien-20-yl acetate (1c) as a yellow wax. The spectroscopic and spectrometric data of 1c are shown in Table S2.

### 4.5. In Vitro Antituberculosis Assay

In vitro anti-TB activity was assessed using the modified MABA described previously [11] on three drug-resistant clinical isolates (MDR [CIBIN/UMF: 15:99], pre-XDR, and XDR), and a drug-susceptible laboratory reference strain (H37Rv, ATCC 27294) [11,23] of *M. tuberculosis* (Table 3). Bacilli were inoculated in tubes with Middlebrook 7H9 broth (Becton Dickinson, NJ, USA) supplemented with 0.2% glycerol (Sigma Aldrich, MO, USA), 10% oleic acid, albumin, dextrose, and catalase (OADC; Becton Dickinson, NJ, USA) incubated at 37 °C in a 5% CO_2_ atmosphere until a turbidity equivalent to that of Mc Farland’s No. 1 standard was achieved with a nephelometer (ATB, bioMérieux, France), and this mycobacterial suspension was diluted 1:50 to obtain a test inoculum close to 6 × 10^6^ CFU/mL immediately before use [11,23].

Both natural and semi-synthetic mulinanes were dissolved with dimethyl sulfoxide (DMSO; Sigma Aldrich, MO, USA) and diluted by two-fold serial dilution in the wells of the microplate containing Middlebrook 7H9 broth (Becton Dickinson, NJ, USA) to be tested at a concentration range of 100.00–0.78 µg/mL. The results were reported MIC and expressed as µM. In each microplate assay, Rifampin (1.00–0.03 μg/mL) for the H37Rv-susceptible strain Ofloxacin (8.00–0.03 μg/mL) for MDR strain, or Clofazimine for pre-XDR and XDR strains (8.00–0.03 μg/mL) (all drugs from Sigma Aldrich, MO, USA) were included as positive controls, while negative controls were compound-free wells. Additionally, the culture medium and DMSO were included as quality and solvent controls, respectively. The MIC was determined by the shift color of the redox-sensitive growth indicator Alamar blue (TREK Diagnostic Systems, Westlake, OH, USA); a well-defined pink color was interpreted as positive bacterial growth, whereas a blue color indicated an absence of growth. The MIC corresponded to the greatest dilution of natural and semi-synthetic mulinanes in which the color shift from blue to pink was not observed. The MIC values were confirmed by direct observation of the number and size of mycobacterial clusters in experimental cultures compared with the untreated controls. Observations were performed with an inverted microscope. Each assay was performed three independent times in duplicate [11].

All compounds were tested for their MBC following the procedure previously described. Briefly, immediately thereafter MABA, 5 µL of the mycobacterial suspensions from the wells corresponding to the ½ MIC, MIC, and 2X MIC values were transferred from the former to a new microplate that contained 195 µL of fresh culture medium per well. The microplates were incubated and developed with Alamar Blue as for MABA. The MBC corresponded to the minimum natural and semi-synthetic mulinanes concentration that did not cause a color shift in cultures reincubated in fresh medium [11].

### 4.6. Calculation of Lipophilicity

The lipophilicity of natural and semi-synthetic mulinanes was calculated in ACD/ChemSketch freeware (ACD/Labs, Toronto, ON, Canada).

### 4.7. Cytotoxicity Assay

The in vitro cytotoxic assay on Vero Cells (ATCC CCL-8) was performed using the Sulforhodamine B Sigma Aldrich, MO, USA) method [23,27]. The natural and semi-synthetic mulinanes were dissolved and sterilized with 100% DMSO and kept at a concentration of 20 mg/mL; dilution of the stock solution with culture medium (Sigma Aldrich, MO, USA) allowed testing of the different compounds at concentrations ranging from 200.00 − 1.56 μg/mL. The results were expressed as the concentration of product that killed 50% of the cells (50% cytotoxic concentration, [CC_50_]). Docetaxel (Sigma Aldrich, MO, USA) and untreated cells were used as positive and negative controls, respectively. All evaluations were performed in triplicate, and CC_50_ values were calculated utilizing GraphPad Prism ver. 5 software (GraphPad Software Inc., La Jolla, CA, USA). The CC_50_ values of natural and semi-synthetic mulinanes were expressed as µM.

### 4.8. Calculation of the SI

The SI was calculated by the ratio of CC_50_ of the tested products on Vero cells/MIC *M. tuberculosis* [28]. 

### 4.9. In Vivo Antituberculosis Activity of Semi-Synthetic Mulinane 1a

The MDR strain of *M. tuberculosis* CIBIN/UMF: 15:99 resistant to Streptomycin, Isoniazid, Rifampn, Ethambutol, and Pyrazinamide (Table 3; [11]) was cultured in Middlebrook 7H9 broth (Becton Dickinson, NJ, USA) with glycerol (Sigma Aldrich, MO, USA) enriched with ADC (albumin, dextrose, and catalase; Becton Dickinson, NJ, USA). After four weeks of growth, the bacteria were harvested in the logarithmic phase at an optical density of 0.400 and a 600-nm wavelength, while the purity was corroborated with Ziehl-Neelsen staining. Bacilli were suspended in phosphate buffer saline (PBS; Sigma Aldrich, MO, USA), aliquoted, and frozen at −70 °C until use. All procedures for the cultivation and use of mycobacteria were carried out in Level 3 biosafety facilities within the Experimental Pathology Laboratory of INCMNSZ. 

For the animal infection model, BALB/c male mice, 8 weeks old and weighing 22 g, were used; the animals were provided by the INCMNSZ Department of Experimental Research and Animal House. Mice were anesthetized with sevoflurane (Sigma Aldrich, MO, USA) vapor for bacterial intratracheal inoculation [29]. Briefly, each mouse was placed on a plate, the incisor teeth were held with a rubber band, a cannula of 22G x1 gauge with a 125 mm blunt tip was introduced through the trachea, and the animal was inoculated with 250,000 CFU of the MDR strain of *M. tuberculosis*, suspended in a volume of 100 µL of PBS. Since mice are not a natural host of *M. tuberculosis*, a high bacterial infecting dose is required to produce a progressive disease [30,31]. Animals were housed in ventilated boxes with air supplied through HEPA filters and were handled under strict Level 3 Animal Biosecurity Protocols by highly qualified personnel. 

To evaluate the therapeutic effect of semi-synthetic mulinane 1a on tuberculous animals, a dose of 0.2536 mg/Kg was used (each mouse received 5.58 µg of compound 1a per dose, dissolved in 0.0948 µL of DMSO and 49.9 µL of injectable water), administered via the intratracheal route under anesthesia with sevoflurane three times a week, from day 60 post-infection and until day 120 within a biosafety level3cabinet. The selection of the appropriate dose was calculated according to the MIC determined in vitro (drug concentration efficient to kill 1 × 10^6^ bacilli) by adjusting the drug concentration to the estimated number of bacilli in the lungs of the mice after two months of infection. The control group only received the vehicle (water plus DMSO). After confirming the anti-TB in vivo activity of 1a, MDR-*M. tuberculosis*-infected mice were treated with 1a (0.2536 mg/kg) by the intratracheal route plus drugs recommended by WHO. Antibiotics were used in suboptimal doses in order to obtain a better evaluation of the eventual synergic activity of the tested compound, in a similar way to the testing of the other therapeutical products using this experimental model [32]. The regimen consisted of 1.1 mg/kg of Amikacin (Sigma Aldrich, MO, USA), 0.55 mg/kg of Ethionamide (Sigma Aldrich, MO, USA), 1.1 mg/kg of Moxifloxacin (Bayer, Leverkusen, Germay), and 1.65 mg/kg of Pyrazinamide (Sigma Aldrich, MO, USA) [33]. These antibiotics were administered daily for 5 days by gavage dissolved in 100 μL of sterile isotonic saline solution, except for Amikacin, which was administered intramuscularly. The intratracheal route of compound 1a administration was selected because a higher therapeutic efficiency has been observed using this route of treatment with diverse compounds [34]. The control group was treated with this antibiotic regime without 1a.

Groups of six mice were anesthetized with sodium pentobarbital (Sigma Aldrich, MO, USA) administered via the intraperitoneal route at a dose of 210 mg/kg and were euthanized by exsanguination at days 30 and 60 post-treatment (90- and 120-days post-infection). Lungs were immediately collected, frozen by immersion in liquid nitrogen, and stored at −70 °C until processing. To determine the bacillary load by means of the CFU count, the lungs were thawed, immersed in PBS-Tween (Sigma Aldrich, MO, USA) 0.05%, and homogenized using the FastPrep24 system (Becton Dickinson, NJ, USA). From the homogenized mixture, serial dilutions were prepared for each sample and 10 µL of each dilution was seeded in a petri dish with Middlebrook 7H10 agar (Becton Dickinson, NJ, USA) enriched with OADC (Becton Dickinson, NJ, USA), and the CFU were counted after 21 days. For histological evaluation, left lungs were fixed by intratracheal perfusion with 10% formaldehyde dissolved in PBS, dehydrated, and included in paraffin. Sections were stained with hematoxylin/eosin and Ziehl-Neelsen.

All procedures were carried out in a cabinet with a class-3 biosafety level. Three independent experiments were performed.

Statistical analysis: The calculation of the sample size (number of mice) was carried out by the following formula based on the serial incidents in the procedures:

X = N/((A/100) × (B/100) × (C/100) …)(1)

where

X = Final number of animals needed or number of animals from which we must start.
N = Minimal statistical number that allows concluding the proposed objectives.
A = 100—a% incidence 1 (deaths from anesthesia during treatment).
B = 100—b% incidence 2 (deaths caused by disease, humanitarian euthanasia).
C = 100—c% incidence 3 (deaths in the control group).
D = 100—d% incidence 4 (deaths caused by anesthesia during infection).

The data obtained were grouped according to each objective, and a 2-way ANOVA analysis of variance and a correction factor for multiple comparisons were performed by Bonferroni test, using GraphPad Prism version 6.01 software (GraphPad Software, Inc., La Jolla, CA, USA).

## 5. Conclusions

The strong anti-TB activity of semi-synthetic derivative 1a (mulin-11,13-dien-20-ol) confirms the importance of the esterification of the natural product for the expression of activity. The semi-synthetic mulinane 1a can be considered as the most promising candidate for future studies because a significant reduction of mycobacterial growth was obtained in both in vitro and in vivo models, while making 1a an important lead molecule and a valuable candidate for future investigations in the discovery of novel antitubercular agents. Future experiments using expanding numbers of candidates and drug-resistant types of *M. tuberculosis* must be carried out to establish the role of this new candidate drug in TB treatment.

## Figures and Tables

**Table 1:** In Vitro antituberculosis activity and lipophilicity of natural and semi-synthetic mulinanes.

Compounds		*Mycobacterium tuberculosis*								Log P
	Type	MDR		Pre-XDR		XDR		Susceptible		
		MIC ^a^	MBC ^a^	MIC ^a^	MBC ^a^	MIC ^a^	MBC ^a^	MIC ^a^	MBC ^a^	
1	N	165.3	165.3	165.3	165.3	82.7	82.7	165.3	165.3	6.37 ± 0.28
**1a**	SS	**2.7**	**2.7**	**2.7**	**2.7**	**2.7**	**2.7**	**5.4**	**5.4**	6.40 ± 0.27
1b	SS	5.4	5.4	5.4	5.4	10.9	10.9	10.9	10.9	6.50 ± 0.30
1c	SS	75.6	75.6	75.6	75.6	75.6	75.6	151.3	151.3	7.34 ± 0.29
2	N	156	156	312	312	312	312	312	312	4.91 ± 0.31
2a	SS	10.2	10.2	20.4	20.4	10.2	10.2	20.4	20.4	4.94 ± 0.30
3	N	157	157	314	314	78.5	78.5	314	314	4.35 ± 0.47
3a	SS	10.3	10.3	10.3	10.3	20.5	20.5	20.5	20.5	4.38 ± 0.41
OFX	Positive controls	1.4	---	---	---	---	---	---	---	---
CZM		---	---	1.1	---	1.1	---	---	---	---
RIF		---	---	---	---	---	---	0.06	---	---

N: natural mulinane; SS: semi-synthetic mulinane; MIC: Minimum Inhibitory Concentration; MBC: Minimum Bactericidal Concentration; OFX: Ofloxacin; CZM: Clofazimine; RIF: Rifampin; MIC and MBC values as expressed as µM. The most bioactive mulinane derivative is written in bold. ^a^ The concentrations of compounds were tested by the two-fold broth dilution method. MIC and MBC determinations were performed three independent times in duplicate with zero variation between experiments.

**Table 2:** Cytotoxic activity and SI of natural and semi-synthetic mulinanes.

Compounds	CC_50_ on Vero Cells	SI
1	18.2 ± 0.79	0.11–0.22
**1a**	**42.2 ± 1.38**	**7.82–15.64**
1b	ND	ND
1c	196.4 ± 8.81	1.29–2.57
2	>624	2–4
2a	>652.8	32–64
3	255.9 ± 21.60	0.82–3.26
3a	>656	32–64
DTX	2.1 ± 0.40	---

SI: Selective Index expressed as a range; CC_50_: 50% cytotoxic concentration; ND: Not Determined. DTX: Docetaxel. The most antituberculosis mulinane derivative is written in bold. CC_50_ values expressed as µM ± SD; SI values expressed as a range.

**Table 3:** Antibiotic resistance profile of *M. tuberculosis* strains.

Microorganism	Drug Resistant Profile
MDR clinical isolate	STR, INH, RIF, EMB, PZA
Pre-XDR clinical isolate	STR, INH, RIF, PZA, LVX, OFX
XDR clinical isolate	STR, INH, RIF, PZA, AMK, KAN, LVX, OFX
H37Rv ATCC 27294	----

STR: Streptomycin; INH, Isoniazid; RIF: Rifampin; EMB: Ethambutol; PZA: Pyrazinamide; LVX: Levofloxacin; OFX: Ofloxacin; AMK: Amikacin; KAN: Kanamycin.

## Data Availability

Data is contained within the article and Supplementary Material. The data presented in this study are available in [insert article or Supplementary Material here].

## Supplementary Materials

The following are available online at https://www.mdpi.com/article/10.3390/metabo11120876/s1, Table S1. The ^1^H (400 MHz, CDCl_3_), ^13^C (100 MHz, CDCl_3_), infrared spectroscopy, and mass spectrometry data for compounds 1a, 1b and 1c, Table S2. The ^1^H (400 MHz, CDCl_3_), ^13^C (100 MHz, CDCl_3_), infrared spectroscopy, and mass spectrometry data for compounds 2a and 3a.
