# Supplementary material for: Activity of Semi-Synthetic Mulinanes against MDR, Pre-XDR, and XDR Strains of Mycobacterium tuberculosis
Source: Metabolites. 2021 Dec 16;11(12):876. doi: 10.3390/metabo11120876 (PMC8703773; doi:10.3390/metabo11120876)
Supplement: Supplementary file 1 [file metabolites-11-00876-s001.zip › metabolites-1476051-supplementary.pdf]

metabolites

MDPI

Supplementary Materials

Activity of semi-synthetic mulinanes against susceptible, MDR, pre-XDR, and XDR strains of *Mycobacterium tuberculosis*

María Alejandrina Martínez-González[1,2], Luis Manuel Peña-Rodríguez[2*], Andrés Humberto Uc-Cachón[1], Jorge Bórquez[3], Mario J. Simirgiotis[3,4], Hugo Brígido Barrios-García[5], Rogelio Hernández-Pando[6], Luis Alberto Loyola[3], Carlos Areche[7], Angel de Jesús Dzul-Beh[1,2], Jorge Alberto Barrios-Payán[6], Dulce Mata-Espinosa[6], Fabiola Escalante-Erosa[2], Karlina García-Sosa[2] and Gloria María Molina-Salinas[1*]

[1] Unidad de Investigación Médica Yucatán, Unidad Médica de Alta Especialidad, Hospital de Especialidades 1, Instituto Mexicano del Seguro Social, 97150 Mérida, Yucatán, Mexico; malejandrinamg_16@hotmail.com; andresuccachon@gmail.com; angeldzulbeh1992@gmail.com; gloria.molinas@imss.gob.mx

[2] Unidad de Biotecnología, Centro de Investigación Científica de Yucatán, A.C., 97205 Mérida, Yucatán, Mexico; lmanuel@cicy.mx; fabiola@cicy.mx; karlina@cicy.mx

[3] Departamento de Química, Facultad de Ciencias Básicas, Universidad de Antofagasta, 02800 Antofagasta, Chile; jorge.borquez@uantof.cl; alberto.loyola@uantof.cl

[4] Instituto de Farmacia, Facultad de Ciencias, Universidad Austral de Chile, 3425 Valdivia, Chile; mario.simirgiotis@gmail.com

[5] Facultad de Medicina Veterinaria y Zootecnia, Universidad Autónoma de Tamaulipas, 87000 Ciudad Victoria, Tamaulipas, Mexico; hbarrios@docentes.uat.edu.mx

[6] Departamento de Patología Experimental, Instituto Nacional de Ciencias Médicas y Nutrición Salvador Zubirán (INCMNSZ), 14080 Ciudad de México, Mexico; rhdezpando@hotmail.com; qcjbp77@yahoo.com.mx; dulmat@yahoo.com.mx

[7] Departamento de Química, Facultad de Ciencias, Universidad de Chile, 1058 Santiago, Chile; areche@uchile.cl

* Correspondence: to: gloria.molinas@imss.gob.mx (GMM-S) and lmanuel@cicy.mx (LMP-R).

Table S1. The ^{1}H (400 MHz, $CDCl_3$), ^{13}C (100 MHz, $CDCl_3$), infrared spectroscopy, and mass spectrometry data for compounds 1a, 1b and 1c.

	**1a**		**1b**		**1c**	
Position	**δ_C, type**	**δ_H (*J* in Hz)**	**δ_C, type**	**δ_H (*J* in Hz)**	**δ_C, type**	**δ_H (*J* in Hz)**
1	24.00	1.07 m, 1.47 m	24.41	1.62 m, 2.10 m	23.48	1.09 m, 1.52 m
2	28.51	1.15 m, 1.62 m	29.10	1.57 m, 1.98 m	29.84	1.13 m, 1.60 m
3	31.81	0.85 m	57.36	1.55 m	57.80	0.85 m
4	48.33	1.40 m	29.10	1.55 m	31.32	1.41 m
5	30.10		61.29		48.29	
6	39.28	1.11 m, 2.06 m	31.83	1.44 m, 2.36 m	31.58	1.13 m, 2.11 m
7	35.07	1.18 m, 1.68 m	36.20	1.44 m, 2.70 m	39.79	1.20 m, 1.62 m
8	49.36		34.89		33.91	
9	54.22	2.17 m	50.78	2.36 m	48.97	2.13 m
10	58.16	1.33 m	54.01	1.77 m	45.36	1.41 m
11	132.86	5.62 dd (12.1, 4.3)	132.15	5.54 dd (6.5, 12.5)	137.09	5.45 m
12	128.02	5.46 m	128.38	5.63 d (12.5)	124.49	5.63 d (12.6)
13	131.81		131.66		137.09	
14	125.73	5.46 m	125.68	5.45 d (7.4)	124.49	5.45 m
15	36.48	1.47 m, 2.73 d (17.2)	40.04	1.44 m, 1.65 m	42.89	1.41 m, 2.72 d (17.4)
16	25.79	1.80 s	26.01	1.79 s	25.20	1.79 s
17	27.39	0.88 s	27.14	0.83 s	28.19	0.87 s
18	23.51	0.86 d (6.4)	23.22	0.85 d (6.0)	21.22	0.83 d (6.4)
19	23.40	1.04 d (6.4)	22.28	0.93 d (6.0)	20.69	1.01 d (6.4)
20	60.29	3.79 dd (11.5, 1.2), 3.57 d (11.5)	207.39	9.83 s	62.46	3.93 d (11.7),4.23 d (11.7)
21					171.60	
22					23.07	2.06 s
IR (KBr):	3,380 (O-H), 2,944, 2,866 cm^{-1}		1,727 cm^{-1}		1,746 cm^{-1}	
LREIMS: *m/z*	288 $[M^+]$		286 $[M^+]$		330 $[M^+]$	

Table S2. The ^{1}H (400 MHz, $CDCl_3$), ^{13}C (100 MHz, $CDCl_3$), infrared spectroscopy, and mass spectrometry data for compounds 2a and 3a.

	**2a**		**3a**	
Position	δ_C, type	δ_H (J in Hz)	δ_C, type	δ_H (J in Hz)
1	25.14	1.06 m, 1.53 m	28.17	1.51 m, 1.90 m
2	28.16	1.32 m, 1.80 m	26.25	1.36 m, 1.90 m
3	59.74	1.17 m	49.44	1.43 m
4	31.49	1.70 m	31.75	1.43 m
5	41.10		58.26	
6	29.59	1.32 m, 2.14 m	29.28	1.12 m, 2.24 m
7	37.23	1.26 m, 1.53 m	33.45	1.09 m, 1.17 m
8	33.93		37.13	
9	41.10	2.01 m	48.82	2.44 d (12.4)
10	51.37	2.01 m	46.91	1.74 m
11	134.57	5.41 m	132.10	5.47 dd (10.9, 1.9)
12	137.03	5.41 m		5.68 dd (10.9, 2.5)
			136.93	
13	71.56		71.12	
14	36.74	1.58 m, 2.01 m	33.93	1.59 m, 1.79 m
15	30.91	0.88 m, 2.69 m	24.31	1.26 m, 2.09 m
16	32.61	1.22 s	29.49	1.20 s
17	28.16	0.93 s	75.18	3.56 dd (10.0, 1.9), 3.85 d (10.0)
18	23.87	0.80 d (6.3)	23.48	0.87 d (6.4)
19	23.85	0.99 d (6.3)	23.40	1.05 d (6.4)
20	59.40	3.39 d (11.6), 3.67 d (11.6)	59.56	3.54 d (11.4), 3.71 d (11.4)
IR (KBr):	3,352 (O-H), 2,943, 2,925, 2,855 cm^{-1}		3,020 (O-H), 2,923, 2,865 cm^{-1}	
LREIMS: *m*/*z*	306 $[M^+]$.		304 $[M^+]$.	
